# 2D Time-Domain Spectroscopy for Determination of Energy and Momentum Relaxation Rates of Hydrogen-Like Donor States in Germanium

**DOI:** 10.1021/acsphotonics.3c01522

**Published:** 2024-03-27

**Authors:** Thomas B. Gill, Sergei Pavlov, Connor S. Kidd, Paul Dean, Andrew D. Burnett, Aniela Dunn, Lianhe Li, Nikolay V. Abrosimov, Heinz-Wilhelm Hübers, Edmund H. Linfield, A. Giles Davies, Joshua R. Freeman

**Affiliations:** † School of Electronic and Electrical Engineering, University of Leeds, Woodhouse Lane, Leeds LS2 9JT, U.K.; ‡ Institute of Optical Sensor Systems, German Aerospace Center (DLR), Berlin 12489, Germany; § School of Chemistry, University of Leeds, Woodhouse Lane, Leeds LS2 9JT, U.K.; ∥ Leibniz-Institut für Kristallzüchtung (IKZ), Berlin 12489, Germany; ⊥ Institut für Physik, Humboldt-Universität zu Berlin, Berlin 12489, Germany

**Keywords:** terahertz, multidimensional spectroscopy, semiconductor impurities, coherent spectroscopy, Rydberg states

## Abstract

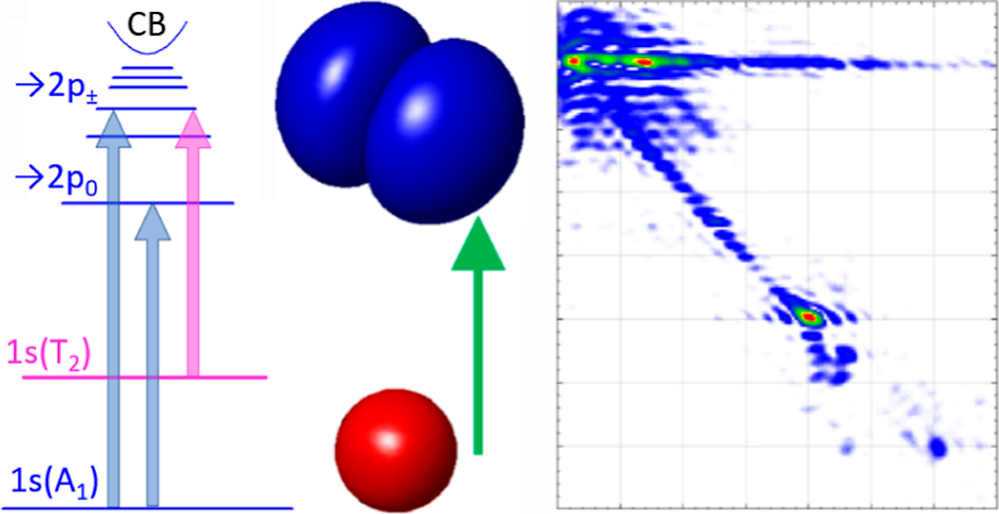

We present measurements of the coherence times of excited states of hydrogen-like arsenic impurities in germanium (Ge:As) using a table-top two-dimensional time-domain spectroscopy (2D-TDS) system. We show that this laboratory system is capable of resolving the coherence lifetimes of atomic-like excited levels of impurity centers in semiconductors, such as those used in solid-state quantum information technologies, on a subpicosecond time scale. By fitting the coherent nonlinear response of the system with the known intracenter transition frequencies, we are able to monitor coherent population transfer and decay of the transitions from the 2p_0_ and 2p_±_ states for different low excitation pulse fields. Furthermore, by examining the off-diagonal resonances in the 2D frequency-domain map, we are able to identify coherences between excited electronic states that are not visible via conventional single-frequency pump–probe or Hahn-echo measurements.

## Introduction

Impurity centers in semiconductors, especially hydrogen-like atoms embedded in group IV crystalline lattices, have long been studied.^1^ Recently they have gained more particular attention in the terahertz (THz) frequency region of the electromagnetic spectrum for use as optically pumped lasers,^2^ ultrafast broadband photoconductive detectors^3^ and for coherent control of atomic orbitals.^4^ Coherent THz control of such impurity states could find applications in quantum technology, where long-lived impurities in silicon are used for single-dopant devices (single-electron),^5^ and in quantum computing (single-spin).^6^ Excited states of hydrogen-like impurities in semiconductors could also be used to build quantum gates implementing collective internal state optical transitions.^7^ For each of these applications, knowledge of the population lifetimes of impurity excited states and their decoherence times is critically important as these define parameters such as the recovery speed of a detector, the optical gain, and the time scale within which atomic states must be manipulated in a quantum computation.

Group IV semiconductors receive the most attention for these applications because of their advanced crystal growth and the doping techniques available, enabling high-quality lattices with precise, targeted doping by substitutional impurity centers. They also benefit from the availability of the most advanced large-scale production and complex device architectures. The decoherence of quantum states in semiconductors is determined mainly by the lattice quality and strength of impurity–phonon interactions, which are the dominant dephasing mechanisms at low crystal temperature and low crystal doping. Lattice-phonon-assisted scattering of electrons bound to excited impurity states is governed by phonon spectra and populations, whose dynamics occur on broad, subpicosecond to subnanosecond time scales. Theoretical predictions of electron–phonon interaction rates in doped silicon are very challenging^8^ and are often far from the experimental observations.^9^ Silicon (Si) doped by hydrogen-like impurities remains a strong candidate for quantum technologies, mainly due to its record for ^28^Si isotope enrichment (0.9999^10^): spin-coherence times exceeding 20 h at temperatures down to several mK were derived from optical spin-pumping pump–probe experiments with ^28^Si/P.^11^ Germanium (Ge) semiconducting crystals hold the records in purification of lattice from electrically active impurities, with densities less than 1 × 10^11^ cm^–3^ achieved.^12^ Germanium crystals exhibit generally weaker electron–lattice interaction than silicon crystals^13^ and interstate energy gaps of their hydrogen-like impurities are significantly lower than the characteristic lattice intervalley phonons.^14^

Measurement of the population lifetimes (*T*_1_) of impurity states can be performed using time-resolved spectroscopy in the frequency domain.^15^ Single-frequency pump–probe (PP) techniques measure the changes in light transmission at the pump frequency by using a weaker probe beam, typically chosen to be in resonance with an impurity intracenter transition. Among the advantages of PP are high sensitivity and high selectivity due to the pump and probe frequencies being resonant with the selected dipole transition in medium. The temporal resolution of PP is limited by the pulse duration of the light source. While for isolated transitions high selectivity is an advantage, in multilevel atomic-like structure of real materials, multiple-path decay is a significant challenge and often simply ignored in the analysis of PP experimental data.

Two-dimensional time-domain spectroscopy (2D-TDS) has been shown to be an effective tool for analyzing nonlinear processes in many materials including multiquantum well systems^16,17^ and identifying photonic and phononic excitation pathways.^18,19^ Furthermore, the technique can simultaneously measure the carrier lifetime (*T*_1_) and polarization lifetime (*T*_2_) in semiconductor multiquantum well systems on ultrashort time scales (<10 fs).^16^ A key advantage of 2D-TDS compared to single-frequency PP is the ability to sample all photoexcited impurity states instantaneously (typical time scales for electron–lattice interactions in cubic semiconductors are longer than 1 ps^20^), while a 2D temporal map gives an overview of different linear and nonlinear phenomena in the medium. This information is a key input for the theoretical modeling of any complex multilevel atomic-like energy spectrum, inherent for all impurity centers in semiconductors. While the temporal resolution of 2D-TDS is also limited by the pulse duration of the light source, the time scale covered is determined by the delay between pulses. Energy resolution, important for resolving atomic states, is determined by the range of times recorded but practically is often limited by reflections in the optical path. The spectral coverage of TDS is typically 0.1–8 THz^21^ which corresponds well to the spectral range of intracenter transitions of hydrogen-like impurities in Ge.^1^ However, reaching high fields at frequencies above 2 THz remains challenging.

**Figure 1:**
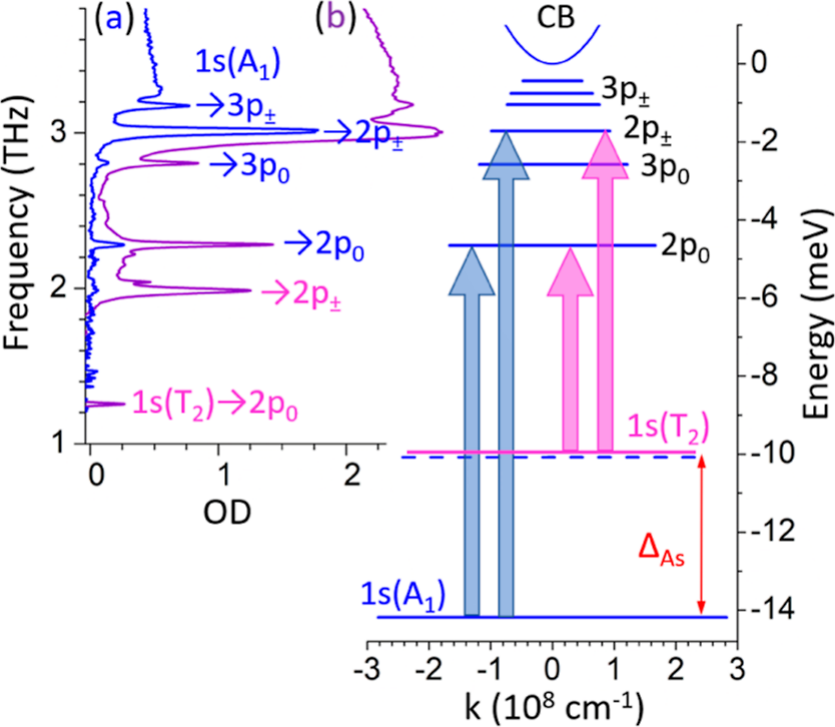
(Left) Absorption spectra of the Ge/As samples measured by FTIR Fourier-transform spectrometer (OD is optical density): [(a) blue] 0.44–mm-thick sample, measured at 5 K with a low-pass 1200 cm^–1^ filter placed in the optical path between the source and cryostat and [(b) purple] 3–mm-thick sample, measured at 20 K, with the low-pass filter removed. Absorption features are labeled with assigned arsenic intracenter transitions. (Right) Energy level diagram of Ge/As, as a function of the wavevector *k*, showing the observed dipole-allowed intracenter transitions, including from the thermally activated 1s(*T*_2_) state. Δ_As_ is the chemical shift of the ground state, CB is the conduction band.

Due to relatively weak electron–phonon interaction in *n-*Ge at low lattice temperatures, 2p donor state depopulation lifetimes have been shown to be in the nanosecond time scale. This time scale is accessible by fast photoconductive spectroscopy, and such experiments revealed relaxation rates of 0.3–0.5 ns^–1^ for the 2p states of antimony donors in compensated germanium (Ge/Sb/B).^22^ These values are consistent with a 1.7 ns lifetime obtained for recombination of free electrons in uncompensated *n*-Ge/Sb using PP at FELIX.^15^ For *n*-Ge/As the relaxation rates of 2p states were estimated to be also ∼1 ns^–1^.^23^ Lifetimes of ∼0.6 ns and ∼0.8 ns were reported for 2*p*_0_ and 2p_±_ states in Ge/As using the NovoFEL free-electron laser at the Siberian Synchrotron and Terahertz Radiation Center (Novosibirsk, Russia).^24^ The temporal resolution was determined by a FEL pulse duration of about 100 ps. Pumping into the 2p_0_ arsenic state was provided by excitation from the donor ground state, 1s(*A*_1_), while pumping to the 2p_±_ state was affected by strong water vapor absorption in the optical path. To avoid this, pumping was achieved via the 1s(*T*_2_) → 2p_±_ transition (the energy scheme of Ge/As is shown in Figure 1b). The relaxation rates were then analyzed in the three-level model of balanced equations for populations in Ge/As. The observed temporal dependencies of the transients had a complex structure indicating multistep relaxation with the shorter time constants estimated to be ∼160 ps, that is, approaching temporal resolution of the pump–probe experiments at this FEL facility.

In this work, we use 2D-TDS based on a table-top amplified laser system to resolve dynamics of electrons bound to excited states of hydrogen-like donor centers. The arsenic donor in Ge, the deepest of the group-V (binding energy *E*_As_ = 14.18 meV (3.43 THz)^1^), is of interest due the proximity of intracenter transitions to the intervalley TA phonon in Ge, providing “partial” electron–phonon resonance, and because of this, potentially the highest relaxation rates of its excited states. The valley-orbit splitting of the arsenic donor state (also known as the chemical shift), the largest from all group-V centers (about 48 K), provides dominant occupation of the ground state at conventional liquid-helium cryogenic cooling systems.

## Sample Details

The germanium crystal was grown in ⟨111⟩ direction by the Czochralski technique with simultaneous doping of arsenic. The arsenic doping concentration is estimated to be 9 × 10^14^ cm^–3^, with compensating gallium acceptors not exceeding 5 × 10^13^ cm^–3^. The samples investigated are 0.44–mm and 3–mm thick in the ⟨111⟩ direction, with optically polished, wedged 10 mm^2^ × 10 mm^2^ facets. We present linear spectra of both samples to maximize the number of visible transitions, while only the thinner sample is used for nonlinear spectroscopy.

Each sample was attached to a coldfinger of a liquid He flow cryostat with silver paint and cooled to 5 K. Absorption spectra (Figure 1) were measured by a Bruker Vertex 80v Fourier-transform infrared (FTIR) spectrometer. The broadband emission source of the spectrometer (>10 000 cm^–1^) activates compensating Ga acceptors producing two weak transitions seen in the spectrum of the thicker, 3 mm sample, (b). These transitions are just visible at 2.04 and 2.22 THz, corresponding to the C- and D-lines of a residual gallium acceptor.

To remove these, an infrared filter (low-pass 1200 cm^–1^) was placed between the source and the cryostat for the measurement of the 0.44 mm-thick sample (Figure 1a). This filter also reduces the thermal load from the FTIR source, removing a detectable population of the lower excited donor state, 1s(*T*_2_), and corresponding transitions with the largest oscillator strengths, namely into the 2p_±_ arsenic state (at ∼1.99 THz) and into the 2*p*_0_ state (at ∼1.25 THz). Again, this is seen in the thicker sample in Figure 1b.

For the 2D-TDS measurements, the 0.44 mm-thick Ge/As sample was clamped to the coldfinger of the cryostat using a copper plate in combination with thermal grease to hold the samples in place and provide thermal coupling. Although cryostat thermometry suggested that a base temperature of ∼8 K was reached, measurements show an initial population in the 1s(*T*_2_) state, indicating that the actual sample temperature may be greater than this. A 1D-TDS measurement was also performed on the sample to verify the transitions were visible using TDS. A summary of this measurement can be found in the Supporting Information.

## Experimental Section

**Figure 2:**
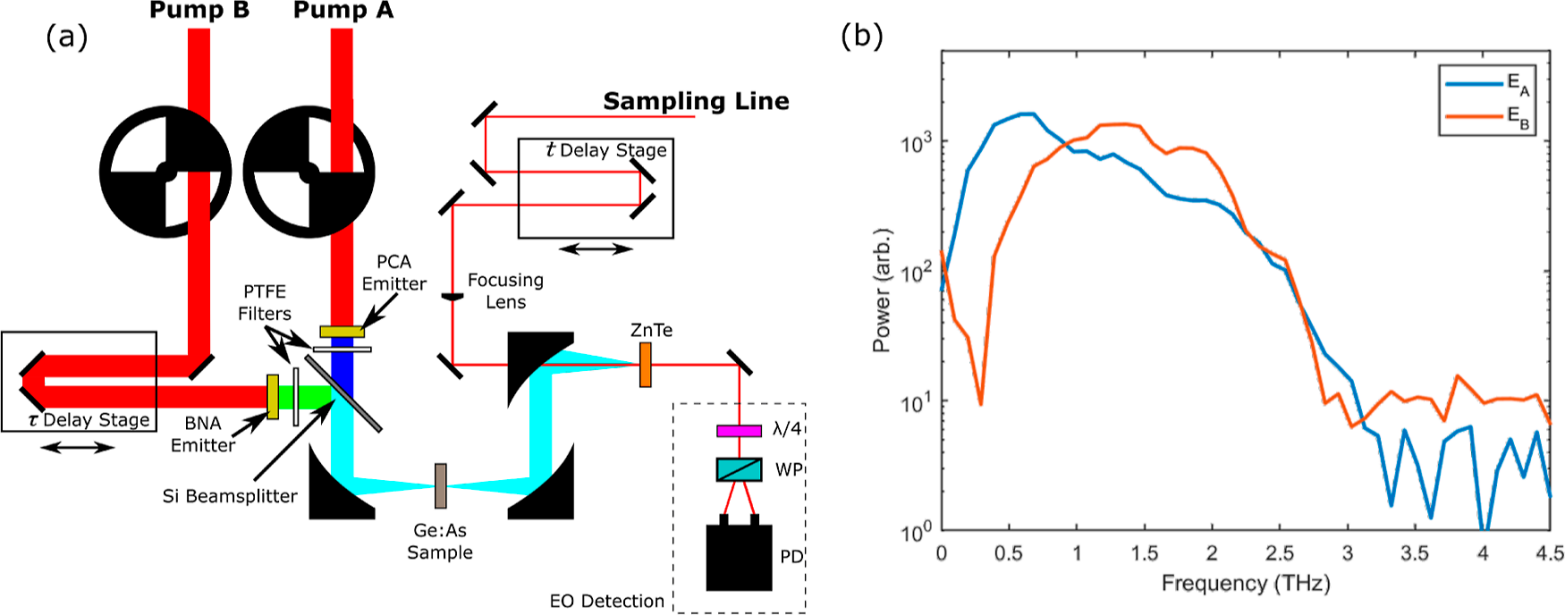
(a) The experimental system used to perform the 2D-TDS measurement. A PCA and a BNA crystal are used to produce the two excitation pulses which are combined using a Si beamsplitter before being focused onto the Ge/As sample using an off-axis parabolic mirror. PTFE filters are placed after the emitters to block the excess IR excitation beam. The detection system consists of nonlinear crystal (ZnTe), quarter-wave plate (λ/4), Wollaston prism (WP) and balanced photodiodes (PD). (b) Spectra of two pump sources used in this work: *E*_A_, generated from a PCA; and *E*_B_, generated from a BNA crystal.

**Figure 3:**
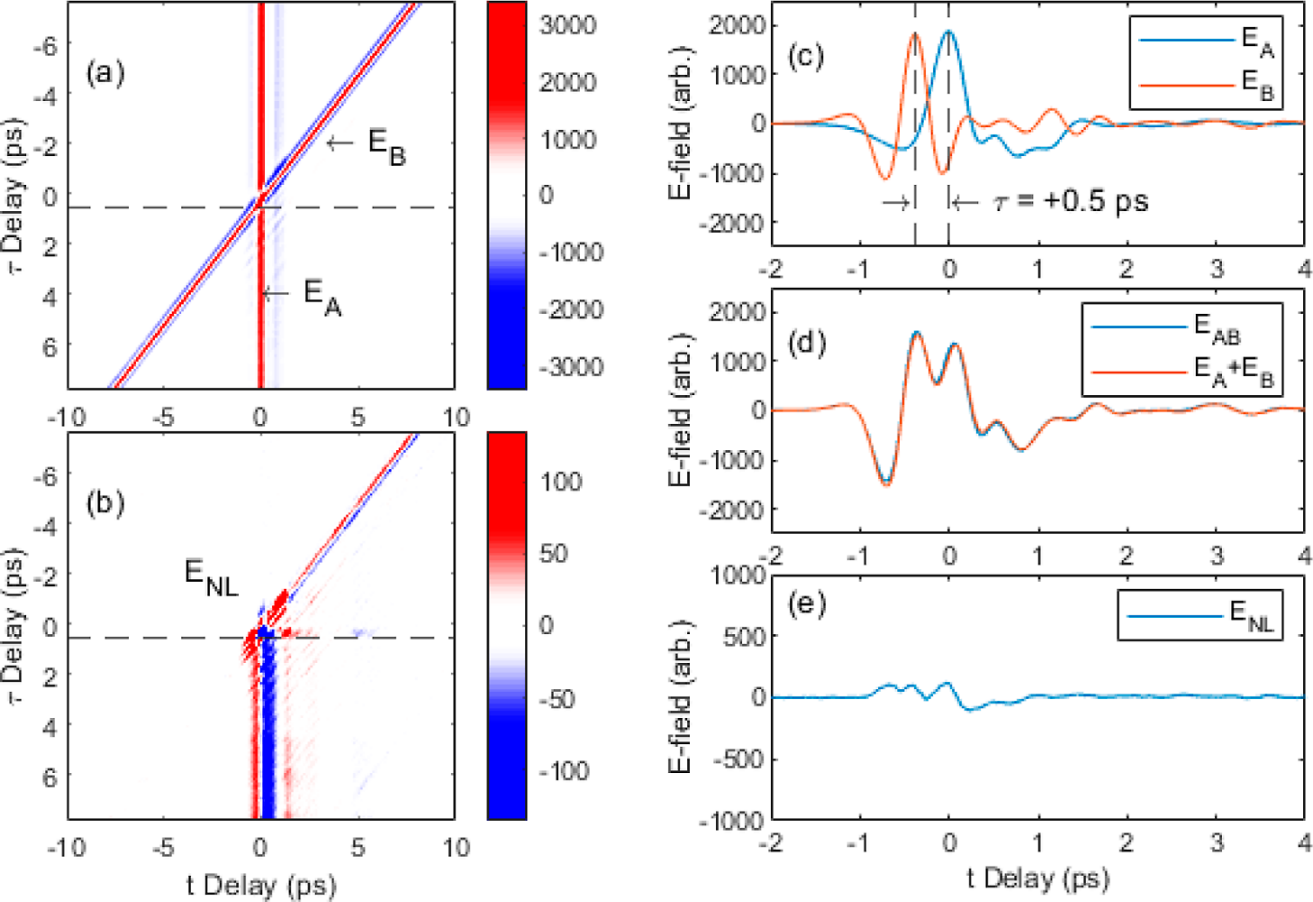
(a) Color plot of *E*_AB_ measured from the 0.44 mm-thick sample for all values of delays, *t* and τ. The dashed line indicates the value of τ = 0.5 ps. (b) Corresponding plot of *E*_NL_, as defined in the text. (c) Signals *E*_A_ and *E*_B_ along the line τ = 0.5 ps. (d) Signal *E*_AB_ and calculated signal *E*_A_ + *E*_B_, the difference between these provides the nonlinear signal *E*_NL_, shown in figure (e).

The two pump pulses required to perform 2D-TDS measurements were generated using a LT-GaAs-on-sapphire photoconductive array (PCA)^25^ and a BNA crystal^26,27^ purchased from Terahertz Innovations LLC, each producing peak electric fields of up to 60 kV cm^–1^, labeled *E*_A_ and *E*_B_ respectively. The pulses generated by the PCA have a larger photon density at low frequency, with a peak emission frequency of 0.6 THz, while the pulses generated from the BNA have a low photon density in this range, with a peak emission frequency of 1.5 THz. Spectra from both pulses is shown in Figure 2b. An amplified laser system generating 40 fs pulses at a center wavelength of 800 nm and at a repetition rate of 1 kHz was used to excite both emitters, with PTFE filters placed after each emitter to block unabsorbed 800 nm light. The pulses were generated using collimated beams and combined using a silicon beamsplitter before being focused onto the sample using a parabolic mirror (see Figure 2a). The pump pulses are chopped at a subharmonic of the laser repetition rate (125 Hz), 90° out of phase to allow the four pulse combinations: *E*_A_ only; *E*_B_ only; *E*_A_ and *E*_B_ together, *E*_AB_; and both blocked, *E*_⌀_, to be acquired for each scan. The delay between *E*_A_ and *E*_B_, denoted as τ, and the delay of the sampling pulse (*t*) are altered throughout the measurement to acquire a 2D signal *E*(*t*, τ), shown in Figure 3a. The electric fields of the THz pulses were detected by electro-optic sampling, using a 1-mm-thick ZnTe crystal, providing a detectable bandwidth of 3 THz. The minimum temporal resolution of the measurements is limited by the pulse width of the sampling beam, which for this system is 40 fs, while the temporal range is limited by the maximum difference in delay that can be achieved. Owing to the time taken to perform 2D-TDS measurements, the temporal resolution and range are set to be lower than achievable to reduce the overall measurement time. For this experiment, a *t*-delay range of ±10 ps with a resolution of 0.01 ps was used, while for the τ-delay, the range was ±8 ps with a resolution of 0.05 ps. The signal acquired from the balanced photodiodes for every pulse is then sent to a boxcar integrator to reduce the noise in the low duty cycle signal. The integrated signal from each laser pulse is then recorded by an ADC, alongside reference signals from the two choppers, allowing the photodiode response at each pulse to be assigned to the correct pump pulse state in postprocessing. The peak field incident on the Ge crystal sample is lower than the peak system capabilities, owing to reflection losses at the cryostat window and sample interfaces. The TPX cryostat window has a refractive index of 1.46^28^ and the Ge crystal has a refractive index of 4.0,^28^ resulting in 77% of the transmitted field reaching the sample.

After the different signal states are acquired and interpolated, the nonlinear response of the sample is calculated using the formula *E*_NL_ = *E*_AB_ – *E*_B_ – *E*_A_,^16^ and is shown in Figure 3b,e. The nonlinear response of the system appears in the lower right portion of Figure 3b, after interaction with both pulses.

## Discussion

**Figure 4:**
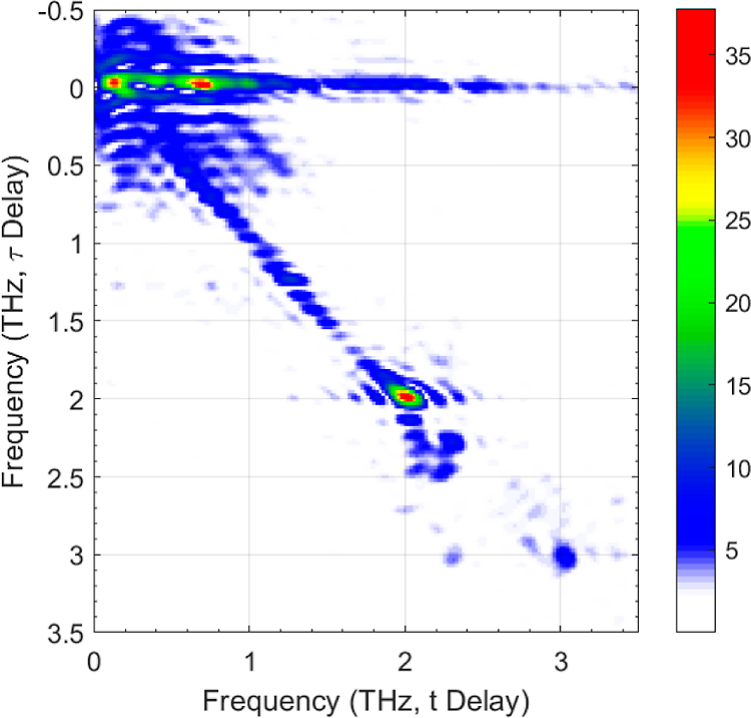
Color plot of . The peak field of *E*_A_ and *E*_B_ are both 23 kV cm^–1^. Peaks in the 2D FFT are seen at the terahertz frequencies: (2,2),(2.3,2.3),(3,3), and (2.3,3). Data has been zero padded to better resolve the features.

Taking the fast Fourier transform (FFT) of the nonlinear signal, , reveals the 2D spectrum of the nonlinear signal, shown in Figure 4. Because of the difference in the spectra between the *A* and *B* pulses, the 2D nonlinear FFT lacks the symmetry seen when these pulses are identical (see, for example refs (16 and 29)). In 2D nonlinear spectra the conventional “pump–probe” signals appear along the line *f*_τ_ = 0 which corresponds to “pump-B–probe-A”, and along the diagonal, *f*_*t*_ = *f*_τ_ which corresponds to “pump-B–probe-A”. Because of the difference between *E*_A_ and *E*_B_, we see a different response in each of these directions.

To explain the nonlinear response to broadband pulses in our sample, we must consider two processes. At low Ge/As lattice temperatures, and before any pulses arrive, carriers in the sample can be considered to be in mainly the 1s(A_1_) state, with some population also in the 1s(*T*_2_) state of the As donor. When a broadband pulse (0.1 to 5 THz) arrives, carriers can be promoted to either an excited state of the hydrogen-like As donor, a bound-to-bound transition (if photon energy, *h*ν < *E*_As_ = 3.43 THz), or, a bound-to-continuum transition (if *h*ν > *E*_As_ = 3.43 THz) if the donor becomes ionized and the electron promoted to the germanium conduction band, where it becomes a free-carrier. Furthermore, measurements on these materials with single frequencies have previously suggested that an “instantaneous” two-step ionization has nonvanishing probability,^9^ where the first photon promotes a ground-state electron to a bound state and a second photon may then excite that excited state electron to the conduction band. For broadband pulses, these two-step excitations may occur in the same pulse at two different photon energies. Where the broadband pulses have a large spectral weight at the lower frequencies, this is likely to be an important process, limited by the absorption rate of the bound-to-bound transitions which are in the 2–3 THz range. Because of the broad bandwidth of our excitation pulses, both are likely to induce a significant population of free-carriers in the sample. However, we also cannot rule out the possibility that scattered light at 800 nm from the amplified laser pulse could generate free-carriers despite the presence of filters to prevent this.

The response observed along the horizontal “pump–probe” line, *f*_τ_ = 0, in the 2D–frequency plot (Figure 4a) shows a strong free-carrier response. This can be understood by recalling that here the *B*-pulse arrives first and the *A*-pulse arrives second. The *B*-pulse, with a larger photon density at higher frequencies (>1 THz) will tend to generate more free-carriers, while the *A*-pulse, with a greater photon density at lower frequencies will be more sensitive to the free-carrier (Drude) response. This pulse sequence amplifies the free-carrier response, making identification of the bound-to-bound transitions along the horizontal, *f*_τ_ = 0, axis difficult (in the time domain, Figure 3b. This is seen as a strong response from 0 ps < τ < 8 ps at *t* ≃ 0 ps). There are also faint ‘echoŝ of this frequency domain response above and below *f*_τ_ = 0 which are an artifact of the finite range of τ used (−8 ≤ τ ≤ 8 ps) and the long-lived free-carriers; note that in the time-domain the strong response around *t* = 0 does not decrease noticeably with increasing τ. This indicates that the lifetime of these free carriers is beyond the time scale of this measurement, ∼ 10 ps.

Turning to the diagonal, *f*_*t*_ = *f*_τ_, where the *A*-pulse arrives first and the *B*-pulse is second (negative τ in Figure 3). Here, the reduced photon density of the *A*-pulse at higher frequencies followed by the low spectral density of the *B*-pulse at lower frequencies acts to suppress the free-carrier response in the nonlinear field *E*_NL_, resulting in bound-to-bound transitions that are more visible. The corresponding time-domain response (τ < 0 region) is weaker because these transitions are more spectrally narrow. In the frequency domain (Figure 4), the narrow bound-to-bound transitions appear as bright regions, mostly along the diagonal *f*_τ_ = *f*_*t*_. While these signatures are faint and limited by the spectral resolution of the TDS measurement, by comparison to high resolution data from Figure 1, they are readily identifiable as the transitions of interest, with features at *f*_τ_ = *f*_*t*_ = {1.25, 1.99, 2.28, and 3.01} THz. These signals correspond to “pump–probe” signals, where the *A*-pulse moves carriers from the ground state(s) to the excited states and the *B*-pulse then experiences a different absorption for each of these transitions, which then appear in *E*_NL_.

We also observe a signal off the diagonal at (*f*_*t*_, *f*_τ_) = (2.28, 3.01) THz. This off-diagonal signal is a signature of coherent correlations between excited states, with an intensity proportional to the dipole matrix element between the states. This allows us to identify coherent coupling between interacting excited states. In this case, it implies coupling between the 2p_±_ and the 2p_0_ levels, either direct or mediated by the 1s(A_1_) ground state. Since the 2p_±_ ↔ 2*p*_0_ transition is forbidden in the first-order approximation, the most likely explanation is coupling via the 1s(A_1_) ground state. The ability of this 2D technique to measure interactions between excited states is a significant advantage over conventional pump–probe studies. We do not observe the corresponding signal at (3.01, 2.28) THz, however this may be a consequence of the low sensitivity of the 1-mm-thick ZnTe detection crystal >2.5 THz because of reduced phase matching between the THz and NIR. This reduction in sensitivity only applies to the *f*_*t*_ – axis since the bandwidth of the *f*_τ_—axis is related only to the delay between the *E*_A_ and *E*_B_ pulses. This reduction in sensitivity is likely to result in any coherence at (3.01, 2.28) THz being below the noise level.

We also note the absence of off-diagonal coupling at (*f*_*t*_, *f*_τ_) = (2.0, 3.0) THz, which suggests no coupling via the 2p_±_ which is likely due to the small population of this state. Furthermore, there is no coherence seen for transition couples that do not share a common state, such as (2.0,2.3) THz.

Finally, we also comment on the lack of four-wave-mixing signals in the 2D FFT. This may because the terahertz field strength used in this work is insufficient to bring these signals above the noise or because they are obscured by the relatively thick sample.

**Figure 5:**
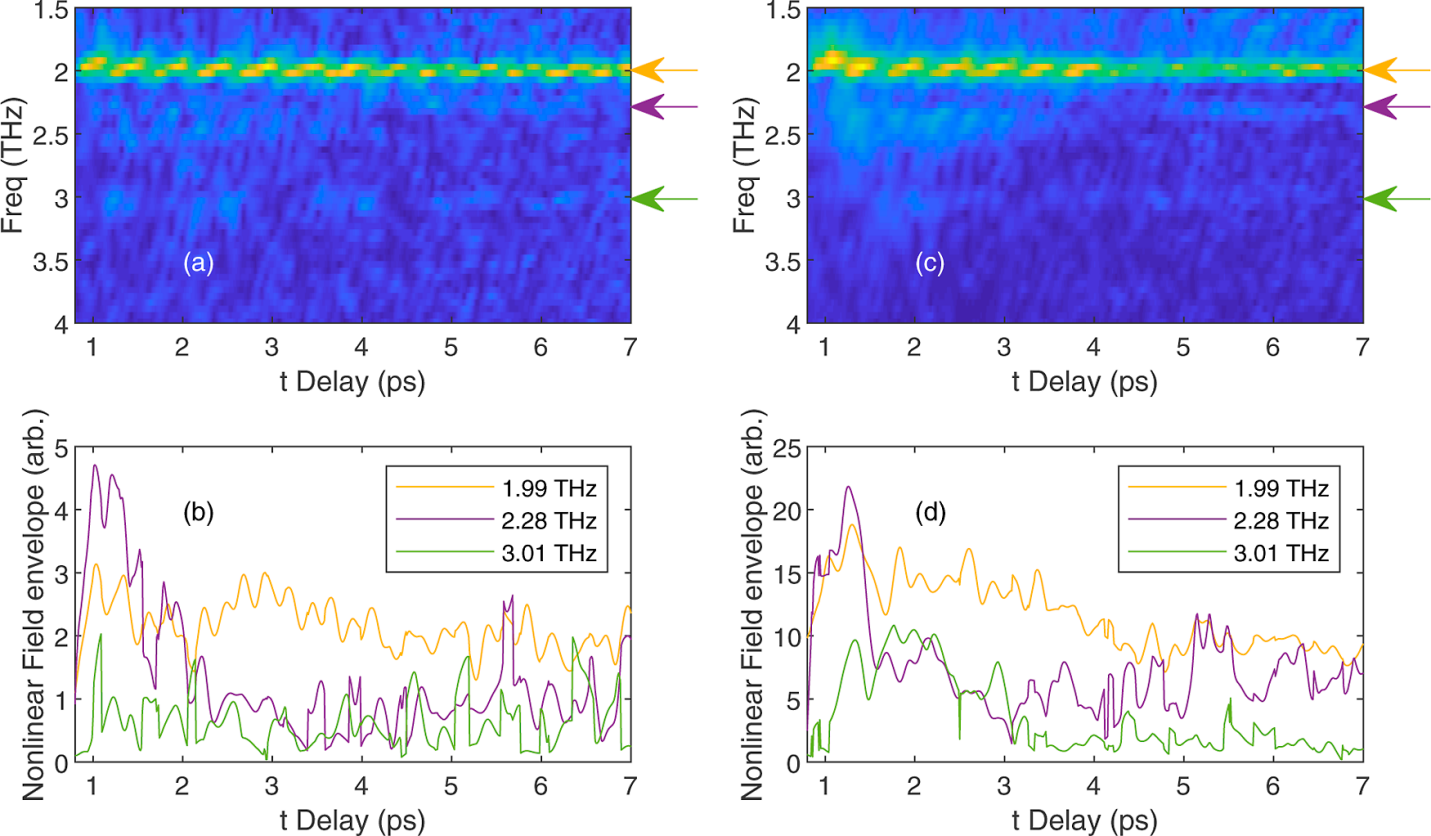
Spectrograms of *E*_NL_ as a function of *t*-delay showing the FFT magnitude for peak fields of (a) 23 and (b) 35 kV cm^–1^. Arrows mark the frequencies 1.99, 2.28 and 3.01 THz. The lower panels show the results of fitting the most prominent frequencies with the expression in eq 1.

After identifying the observed transitions, we return to the nonlinear time-domain data, Figure 3b, to analyze the temporal response of these transitions. In the nonlinear time-domain the oscillation of the polarization appears along the *t*-axis, while the coherent system memory appears as an oscillating signal along the τ-axis.^16^ In Figure 5a,c we plot spectrograms for peak fields of 23 and 35 kV cm^–1^ by taking FFTs along the only the τ-direction for all values of *t*. Here, we are able to identify the same frequencies as those in the 2D FFT above, marked by arrows. Examining the amplitude at these frequencies as a function of *t*-delay, we observe a rapid oscillation at each frequency corresponding peaks on the diagonal of the 2D FFT, above. Furthermore, we observe some slowly varying *t*-dependence. We parameterize the time dependence by returning to the 2D time domain data (Figure 3b) and fitting curves at each value of *t*. This allows for the population decay and dephasing time of the transition to be acquired by fitting with the function
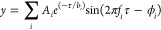
1where the index *i*, refers to the different frequency components, *A*_*i*_ is the amplitude of each component, *b*_*i*_ is the decay time, *f*_*i*_ is the frequency of the component, and ϕ_*i*_ is the phase. We attempt to fit three components with frequencies fixed at [*f*_1_, *f*_2_, *f*_3_] = [1.99, 2.28, 3.01] THz, corresponding to the transitions observed in the high resolution spectra (Figure 1). To improve the signal for fitting, we perform a high-pass filtering (*f*_cut-off_ = 1.0 THz) to remove the low-frequency response of the ionized free-carriers and any slow change in response due to population, which appears as an offset along the *y*-axis in our time-domain signals. In this sample, the population lifetime, *T*_1_, has been measured to be > 600 ps,^24^ much longer than the measurement time.

**Figure 6:**
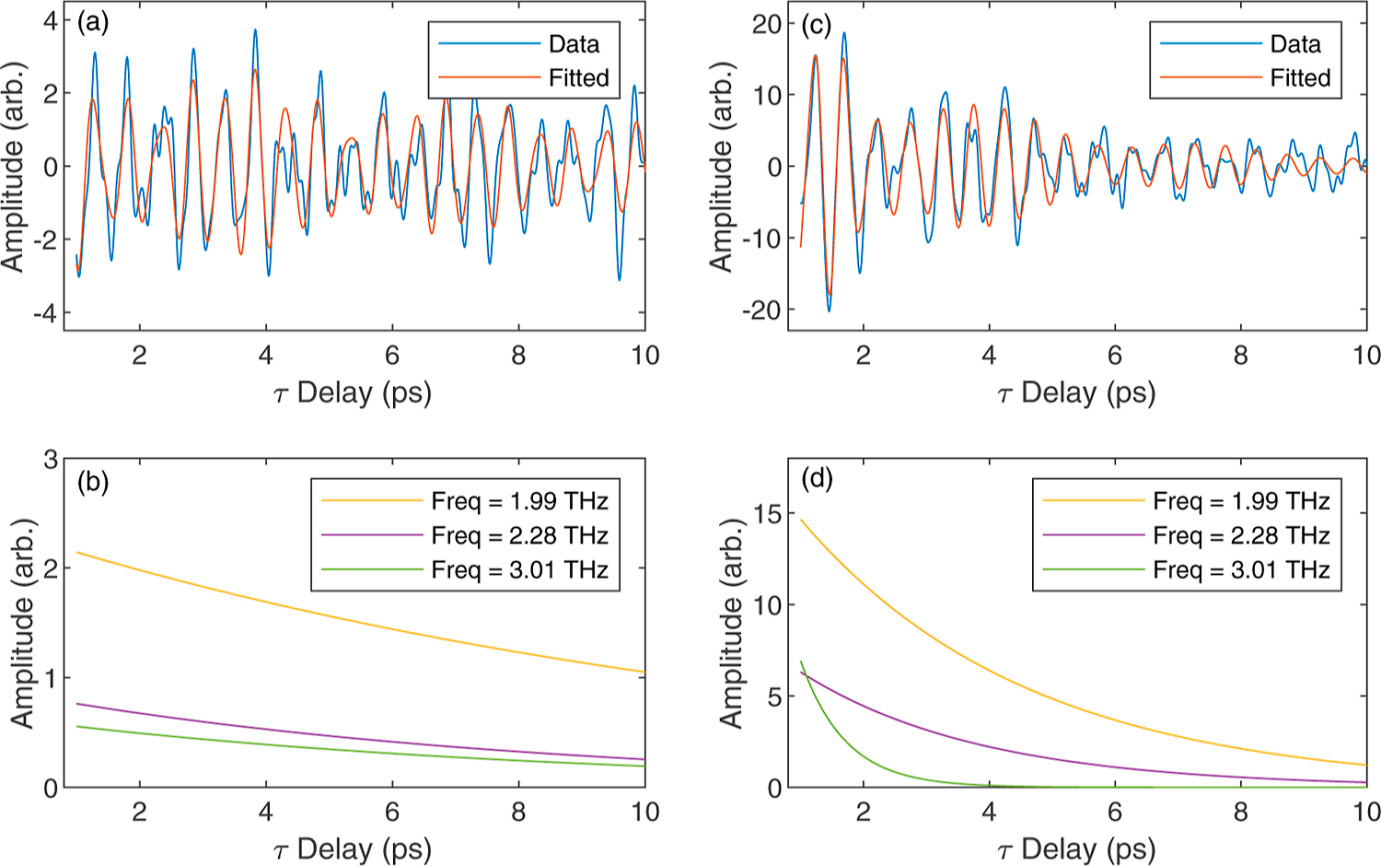
(a) The nonlinear field, *E*_NL_(*t* = 2.4 ps, τ), fitted with eq 1 with peak fields of *E*_A_ = *E*_B_ = 23 kV cm^–1^ and (b) the corresponding amplitude of each frequency component, . (c) The nonlinear field, *E*_NL_(*t* = 2.4 ps, τ), fitted with eq 1 with peak fields of *E*_A_ = *E*_B_ = 35 kV cm^–1^ and (d) the corresponding amplitude of each frequency component.

Slices taken along the τ-axis for *t* = 2.4 ps and peak fields of 23 and 35 kV cm^–1^ are shown in Figure 6a,c; the amplitudes of each frequency component, from fitting, are shown in the corresponding Figure 6b,d. The data shows a multifrequency oscillation, with an amplitude that decays over time. The above equation provides a good fit to the measured data and is able to reproduce the multifrequency oscillation. The fitting starts from 1 ps after the pulse arrives to avoid the region where the two pulses are overlapping. As expected from the nonlinear spectra, the strongest frequency component is at 1.99 THz with associated decay times of *b*_1_ = 12.6 ps and *b*_1_ = 3.6 ps for Figure 6b,d respectively. Amplitudes at 2.28 THz show a slightly faster decay with time constants of 8.2 and 2.9 ps. The component at 3.01 THz appears to have a slightly weaker contribution to the signal and decay times of 8.5 and 0.7 ps, respectively.

While only one slice along τ is presented in Figure 6 for two different pulse energies, this fitting can be done for all values of *t* > 0. In Figure 5b,d, we present a summary of this fitting for each peak field, showing the amplitude of each frequency component. The amplitudes for the lower field, 5b, show that the transition at 2.28 THz first decays rapidly, but appears to increase again after ∼5 ps. The 1.99 THz transition exhibits a steady exponential decay with an associated time of *T*_2_ = (18 ± 2) ps. Turning to the higher excitation field, (5c) the results are similar; while the 1.99 THz component is the strongest, its decrease over time has an associated time of *T*_2_ = (8.8 ± 0.4) ps. Again, the 2.28 THz component first decreases rapidly, between 1 to 2 ps where there is also an associated rise in the 3.01 THz component around 1.5–3 ps, this then gives way to a return of the 2.28 THz component at times >4 ps. This coherent oscillation between the components at 2.28 and 3.01 THz confirms the observation of the off-diagonal peak at this frequency in the 2D nonlinear FFT (Figure 4). While difficult to assign lifetimes to frequency components that oscillate in time, we are able to get an approximate coherence lifetime, *T*_2_, by fitting the nonlinear field envelope of each frequency component; a summary of this fitting is shown in Table 1.

**Table 1:** Table of Lifetimes for the Observed Transitions Obtained from Fitting the Nonlinear Field Envelope^a^

transition	frequency THz	12 kV cm–^1^	23 kV cm–^1^	35 kV cm–^1^	46 kV cm–^1^
1s(*T*_2_) → 2p_±_	1.99	(5.4 ± 0.3)ps	(18 ± 2)ps	(8.8 ± 0.4) ps	(3.1 ± 0.1) ps
1s(*A*_1_) → 2p_0_	2.28	-	(3.0 ± 0.3)ps	(7.6 ± 1.0) ps	
1s(*A*_1_) → 2p_±_	3.01	-		(2.7 ± 0.2) ps	

a The error is taken as the 95% confidence interval from the fit.

**Figure 7:**
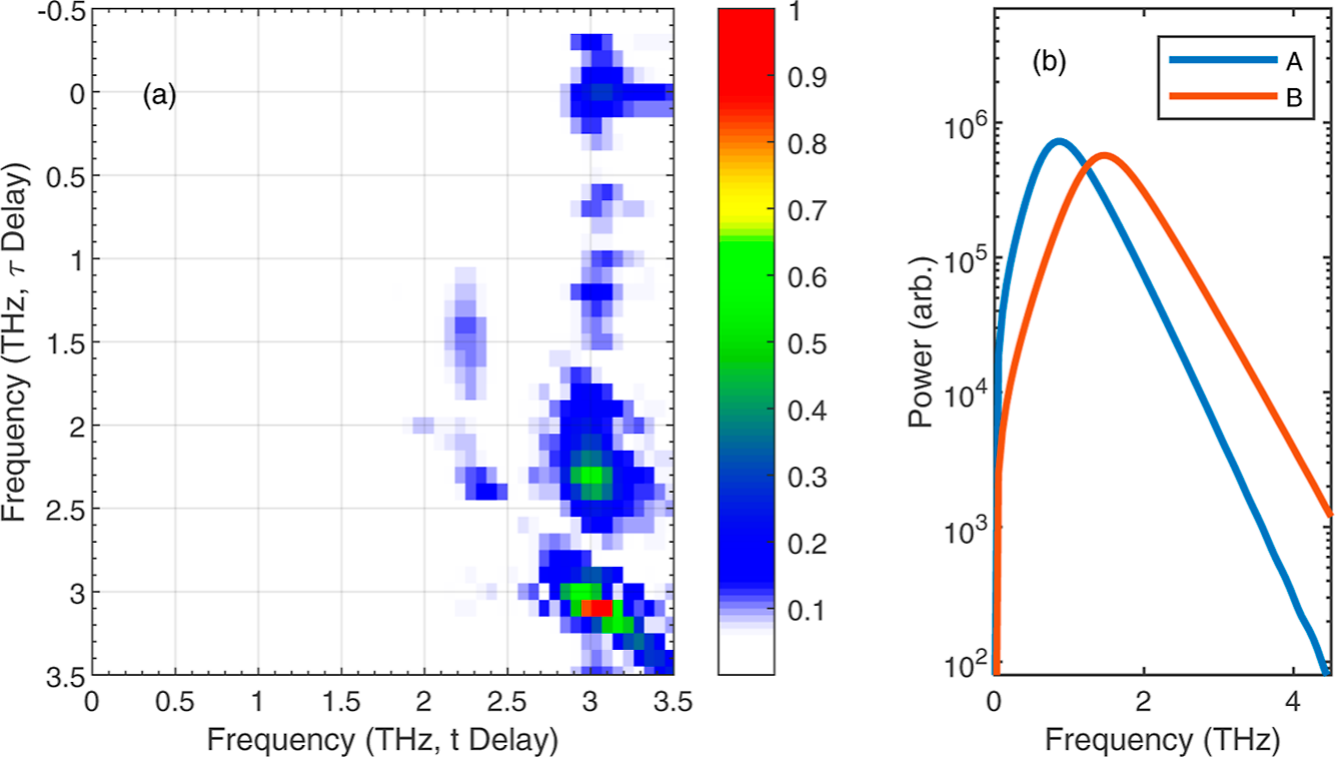
Simulated 2D spectroscopy using Maxwell–Bloch 4-level simulation. (a) Color plot of . The peak field of *E*_A_ and *E*_B_ are both 2 kV cm^–1^. (b) The spectrum at input of each pulse.

To aid the interpretation of our results we have also simulated the 2D spectroscopy measurement using a 4-level Maxwell-Bloch model; further details of the simulation are given in the Supporting Information. The simulation was performed using the same state energies as shown in Figure 1 and dipole matrix elements calculated from Clauws et al.^30^ The pair of excitation pulses used in the simulation were matched with the experiment, however the limited bandwidth of the experimental detection was not accounted for. The simulation describes a closed system where the sum of the 4-level populations remains constant; therefore it does not include thermally activated carrier escape to the continuum and does not account for photoionization to the continuum or any other states. This accounts for the lack of signal in the simulated 2D FFT at frequencies <1 THz. Simulations were performed for peak fields of 1, 2, and 5 kV cm^–1^, with results from the 2 kV cm^–1^ simulation presented in Figure 7. The simulation is able to reproduce the key features of the experimental data. These results are obtained for relatively modest excitation fields with pulses that only cause a small rotation of the Bloch vector, less than , where a π-pulse corresponds to full population inversion. Nevertheless, we are able to extract the *T*_2_ time from the simulated data using the same technique used to analyze the frequency components of the nonlinear 2D time-domain data. We also observe an oscillating decay in the amplitude of the 2.28 THz component, further details of this are given in the Supporting Information. Four-wave mixing signals are visible in the simulated 2D FFT away from the diagonal and off-diagonal; however these are relatively weak, explaining why they are not visible in the experimental data. A difference between the simulation and the experimental data is the coherence at (3.01, and 2.28) THz, which is not visible in Figure 4. In the simulation this appears to be stronger than the coherence at (2.28, 3.01) THz. The explanation for this is likely to be the steep roll-off in sensitivity after 2.5 THz of the 1-mm-thick ZnTe crystal used for EO-sampling in the experiment.

## Conclusions

We have demonstrated the use of a benchtop 2D time-domain spectroscopy system to probe and understand a hydrogen-like atom trapped in a solid host. This method provides several important advantages over a single frequency pump–probe, which is typically achieved using a free-electron laser source. The two key differences with our technique are (1) the broad-band pulse and (2) the coherent detection. We have demonstrated how the broadband pulse allows us to obtain a 2D spectrum and have used this to observe both “pump–probe” signals, where the pump and probe signatures appear at the same frequencies, and to analyze coupling between excited states of the system. Here, we observed coupling between transitions at 2.28 THz and 3.01 THz, in agreement with expected dipole selection rules. Furthermore, we were able to observe a coherent oscillation between states by fitting the nonlinear time-domain response with a sum of decaying sine waves. By fixing the frequencies of these waves at the observed transition frequencies, we are able to plot the coherent response of each transition on a subpicosecond time scale.

In future studies of such materials, it will be possible to combine this 2D time-domain spectroscopy technique with high-pass or band-pass filters to avoid low frequency excitation or even selectively excite a single transition. Experimental systems could also be easily adapted to extend the delay to observe longer-lived coherences than measured in this study. Finally, it is worth noting that while the bandwidth of the excitation pulses and detection was limited to around 3 THz in this work; this bandwidth can be easily increased by replacing the THz pulse generation crystal and the electro-optic detection nonlinear crystal, thereby opening a wider range of dopants in germanium and silicon.

## Data Availability

The data associated with this paper are publicly available from the University of Leeds Data Repository at https://doi.org/10.5518/1486.

## Supporting Information Available

1D spectra from the sample; 2D spectra from the sample at all measured fields; details of the Maxwell–Bloch model and parameters used; analysis of simulated data using the same techniques as the experimental data (PDF)
