# Supplementary material for: 2D Time-Domain Spectroscopy for Determination of Energy and Momentum Relaxation Rates of Hydrogen-Like Donor States in Germanium
Source: ACS Photonics. 2024 Mar 27;11(4):1447–55. doi: 10.1021/acsphotonics.3c01522 (PMC11027176; doi:10.1021/acsphotonics.3c01522)
Supplement: Supplementary file 1 — ph3c01522_si_001.pdf [file ph3c01522_si_001.pdf]

Supplementary information: 2D Time-Domain Spectroscopy for Determination of Energy and Momentum Relaxation Rates of Hydrogen-Like Donor States in Germanium

Thomas B. Gill,*,† Sergei Pavlov,‡ Connor S. Kidd,† Paul Dean,† Andrew D. Burnett,¶ Aniela Dunn,¶ Lianhe Li,† Nikolay V. Abrosimov,§ Heinz-Wilhelm Hübers,‡,‖ Edmund H. Linfield,† A. Giles Davies,† and Joshua R. Freeman*,†

†*School of Electronic and Electrical Engineering, University of Leeds, Woodhouse Lane, Leeds, LS2 9JT, UK*

‡*Institute of Optical Sensor Systems, German Aerospace Center (DLR), 12489 Berlin, Germany*

¶*School of Chemistry, University of Leeds, Woodhouse Lane, Leeds, LS2 9JT, UK*

§*Leibniz-Institut für Kristallzüchtung (IKZ), 12489 Berlin, Germany*

‖*Institut für Physik, Humboldt-Universität zu Berlin, Berlin, Germany*

E-mail: t.b.gill@leeds.ac.uk; j.r.freeman@leeds.ac.uk

List of Figures

S1 Normalised frequency response of the sample when measured using one-dimensional time-domain spectroscopy. 2
S2 Colour plot of $\tilde{E}_{\mathrm{NL}}(f_t, f_\tau)$ at four measured peak field strengths. 3
S3 Simulated time domain response of the 4-level Maxwell-Bloch model. 6
S4 Results of fitting to a 4-level Maxwell-Bloch model. 7
S5 Results of fitting to a 2-level Maxwell-Bloch model. 8

List of Tables

S1 Parameters used in equation 1 simulate each pulse, ‘A’ and ‘B’ 4
S2 Material parameters used for the Maxwell-Bloch simulation. 5

Total pages: 9

One-Dimensional Spectra

Figure S1 shows results from a separate one-dimensional time domain spectroscopy measurement of the sample measured in this work, using a setup similar to the 2D-TDS experimental setup, but with a single pump, generated by a PCA. The sample was mounted in the same cryostat using the same technique as used in the 2D-TDS measurement. The sample was cooled to 10 K before time domain traces were obtained using a peak pump field of 14 kV cm^{-1}. To isolate the response of the bound impurity states, a second time domain measurement was performed immediately after the initial measurement with the sample warmed to 100 K. At this higher temperature, the hydrogen-like donor states are fully ionised. From this measurement, strong absorption responses can be seen at 2.28 THz ($1s(A_1) - 2p_0$) and 2.83 THz ($1s(A_1) - 3p_0$), whilst weaker responses can be seen at 3.0 THz ($1s(A_1) - 2p_\pm$) and 2.0 THz ($1s(T_2) - 2p_\pm$). The large 'transmission' response seen at low frequencies is a by-product of normalising the data to a high temperature measurement, as at higher temperatures, low frequency photons will be absorbed by free carriers in the bulk Ge.

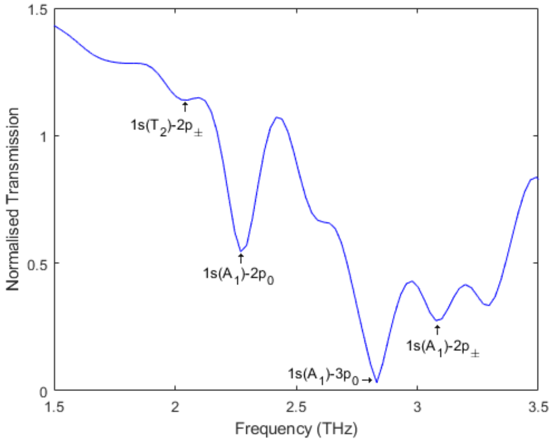

Figure S1: Normalised frequency response of the sample when measured using one-dimensional time-domain spectroscopy. Probe pulses generated from a PCA with a peak field of 14 kV cm^{-1}. Sample measured at 10 K and normalised using data from an identical measurement with the sample warmed to 100 K.

Two-Dimensional Spectra

Figure S2 shows 2D spectra for all peak fields measured. At the lowest peak field of $12\,\mathrm{kV\,cm^{-1}}$, figure S2(a), the features at (1.99,1.99)THz and (3.01,3.01)THz are clearly visible. As the peak field is increased to $23\,\mathrm{kV\,cm^{-1}}$ (fig. S2(b)), the peak at (3.01,3.01)THz reduces in intensity, but a faint peak at (2.28,2.28)THz appears, together with an off-diagonal peak at (2.28,3.01)THz. This off-diagonal peak is discussed in the main text. As the peak field is increased to $35\,\mathrm{kV\,cm^{-1}}$ and $46\,\mathrm{kV\,cm^{-1}}$, figs. S2(c) and (d), the isolated peaks become less distinct and a broadband response, attributed to free-carriers, begins to dominate the 2D spectrum.

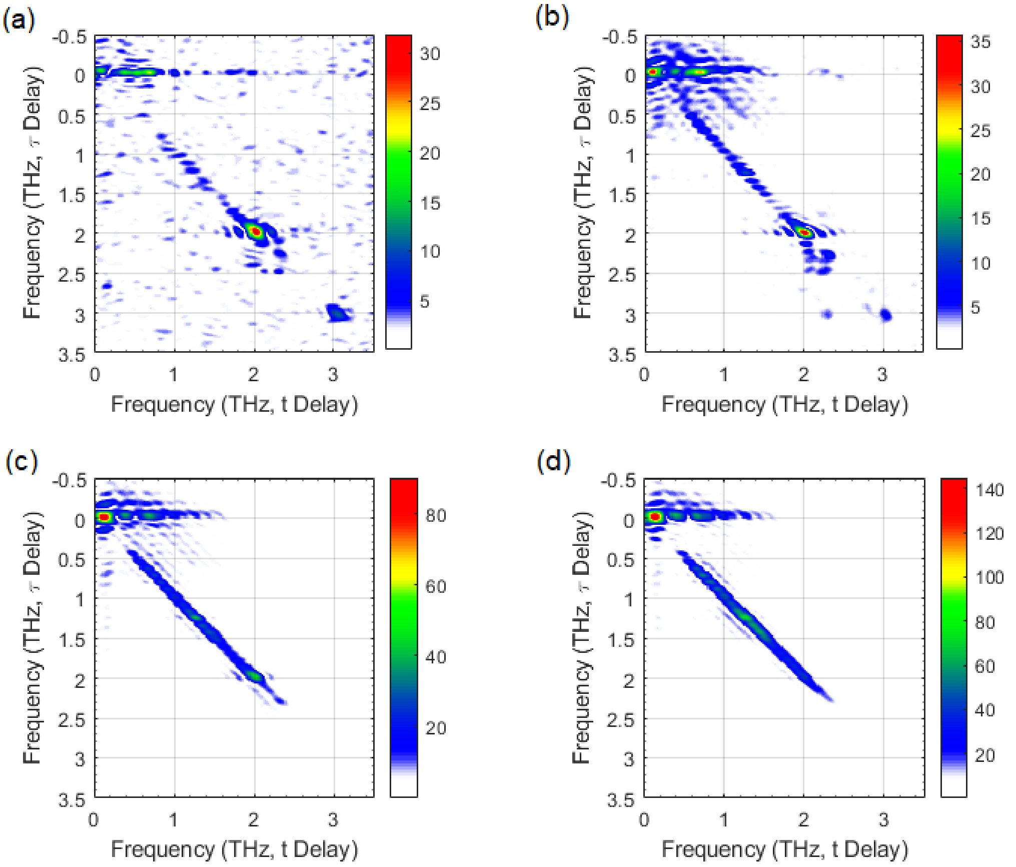

Figure S2: Colour plot of $\tilde{E}_{\mathrm{NL}}(f_t, f_\tau)$ for peak fields of (a) $12\,\mathrm{kV\,cm^{-1}}$, (b) $23\,\mathrm{kV\,cm^{-1}}$, (c) $35\,\mathrm{kV\,cm^{-1}}$, and (d) $46\,\mathrm{kV\,cm^{-1}}$. Data has been zero padded to better resolve the features.

Maxwell-Bloch Model

The 2D time-domain response is simulated using a four-level Maxwell-Bloch model, solved numerically using the finite-difference time-domain (FDTD) method, described in Ref.[1] and extended to four levels. The 'A' and 'B' pulses used as inputs for the simulation are found by fitting the experimental measured pulses. We approximate the experimental pulses with the function

$$E(t) = E_0 \cos(2\pi f_p(t))\text{sech}\left(1.76\frac{t-t_c}{t_p}\right). \tag{1}$$

The description of each parameter and the values for the A and B pulses are given in table S1. In an analogous way to the experiment, the simulation must be run for each value of delay, τ. The electric fields that transit the 4-level medium are recorded at the end of the medium and are then analysed using the same methods as the experimental data. The finite length of the sample, and the need to remove reflections from the sample in the data before taking the Fourier transform, limits the resolution of the simulated data to the same as the experimental data.

Table S1: Parameters used in equation 1 simulate each pulse, 'A' and 'B'

Parameter	Symbol	A	B
Field amplitude	E_0	$2\,\text{kV cm}^{-1}$	$2\,\text{kV cm}^{-1}$
Carrier frequency	f_p	741 GHz	1.37 THz
Carrier offset	t_c	-0.028 ps	-0.042 ps
Pulse length	t_p	0.55 ps	0.45 ps

We formulate the Maxwell Bloch equations, for an n-level system, where the field propagates in the $z-$direction:

$$\partial_t H_y = -\frac{1}{\mu_0}\frac{\partial E_x}{\partial z} \quad (2)$$

$$\partial_t E_x = -\frac{1}{\varepsilon}\frac{\partial H_y}{\partial z} - n\mathcal{P}U \quad (3)$$

$$\partial_t U = \left(\left(E_x\mathcal{A} + \bar{\mathcal{A}}\right) \odot \mathcal{M}\right) U - dU_s \quad (4)$$

Where H_y and E_x are the magnetic and electric fields in the $y-$ and $x-$ directions, respectively. The vector U contains both the real diagonal elements and complex off-diagonal elements of the density matrix. For the present case of a 4-level system, there are four diagonal elements leading to three population differences, and six complex off-diagonal elements in the Hermitian matrix, leading to 12 real and imaginary parts, and a total of 15 elements in the real vector U. N is the number of 4-level systems and the 15-element vector $\mathcal{P}$ contains coefficients dependent on the dipole matrix elements and energies of the system. To simplify the computation, the electric field, E_x, is factored out from the coefficient matrix using the matrix $\mathcal{A}$, containing only zero or one for each element, and the logical NOT() of this, $\bar{\mathcal{A}}$. The vector dU_s contains constant decay terms. This set of equations is then solved using the FDTD predictor-corrector method, detailed in Ref.[2] The material parameters used for the simulation are given in table S2.

Table S2: Material parameters used for the Maxwell-Bloch simulation.

Parameter	Symbol	Value
Relative permittivity	ϵ_r	12.9
Population lifetime	T_1	50 ps (all levels)
Coherence lifetime	T_2	2 ps (all levels)
Oscillator density	n	$1 \times 10^{14}\mathrm{cm}^{-3}$
Temperature	T_0	20 K
Energy levels (relative to ground state)	f_n	{1.05, 2.3, 3.0} THz
Cavity length	L	440 µm

The dipole matrix elements used for the simulation, calculated from Ref.[3] and in units

of nanometers, are:

$$z = \begin{bmatrix} - & 0 & 0.6 & 1.6 \\ - & - & 0.6 & 1.6 \\ - & - & - & 0.5 \\ - & - & - & - \end{bmatrix}$$

The dipole elements are given in matrix form, with states labelled 1–4 starting from the ground state. For example, the dipole matrix element between the ground state (1) and the first excited state (2), 1.05 THz above the ground state, is $z_{21} = 0$. The matrix elements are chosen to simulate the energy structure of the arsenic atom in germanium so that the levels $\{1s(A_1), 1s(T_2), 2p_0, 2p_\pm\}$ are assigned to the simulation levels {1,2,3,4} with energies at {0,1.05, 2.3, 3.0}THz.

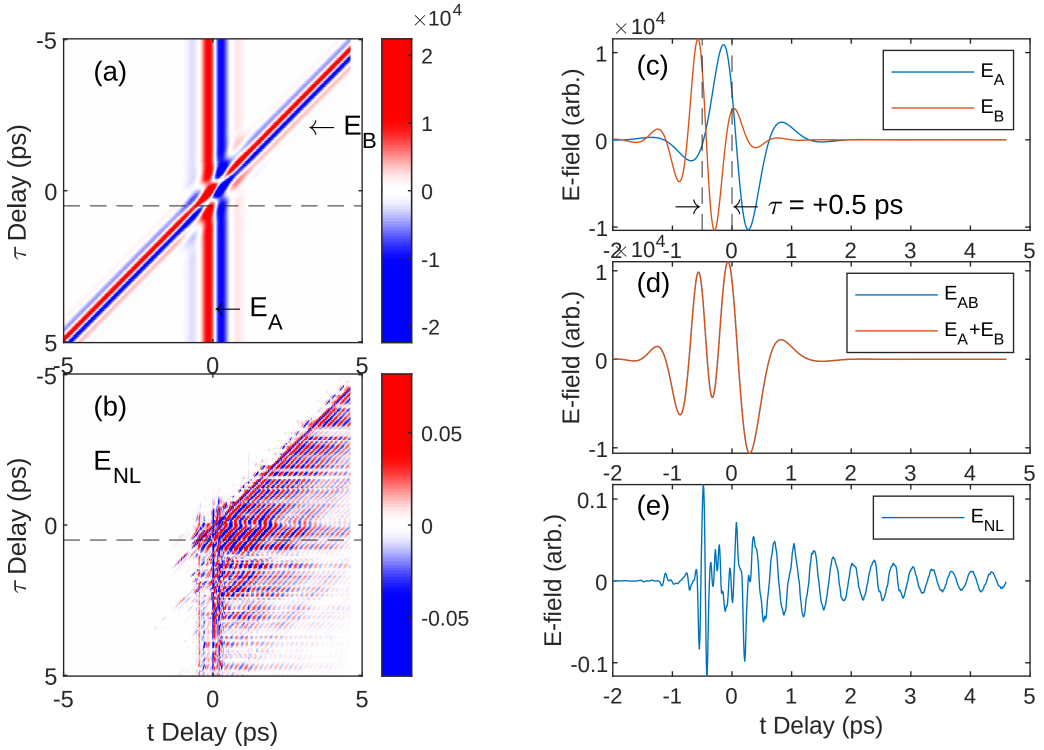

Figure S3: Simulated time domain response of the 4-level Maxwell-Bloch model.(a) 2D time-domain response of E_{AB} signal and (b) corresponding nonlinear signal, E_{NL}. (c)-(e) Shows the response at a delay between pulse A and B of $\tau = 0.5\,\mathrm{ps}$, for (c) pulses E_{A} and E_{B}, (d) pulses E_{AB} and $E_{\mathrm{A}}+E_{\mathrm{B}}$ and (e) E_{NL}.

Results from the simulation are shown in figure S3. The simulation process for 2D nonlinear time-domain spectroscopy proceeds in the same way as the experiment; the simulation is run for 3 different input pulses 'A pulse only', 'B pulse only' and 'A+B pulses'. The nonlinear signal is then calculated from $E_{\mathrm{NL}} = E_{\mathrm{AB}} - E_{\mathrm{A}} - E_{\mathrm{B}}$. The Fourier transformed, frequency-domain equivalent is shown in fig. 6 in the main text.

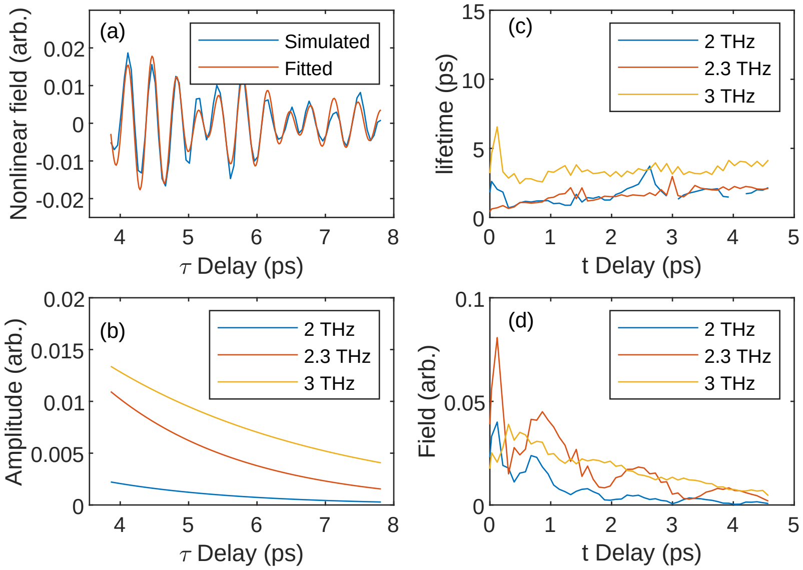

Figure S4: (a) Fitting a slice along the τ-axis for a fixed value of t of the nonlinear signal generated by the 2D-TDS simulation with the 4-level Maxwell-Bloch model. (b) Amplitude of each frequency component from the same fit. (c) The lifetime of each frequency component extracted from each fit, and (d) The maximum value of the field obtained from fitting along the τ direction for each value of t.

We have used the same fitting procedure as the main text to analyse the results obtained from the 4-level Maxwell-Bloch model, with the results shown in fig. S4. A slice along the τ-axis at fixed t and corresponding fit (using eqn. 1 from the main text) are shown in fig. S4(a) and (b). As expected, the fit gives good agreement with the data, clearly indicating the multiple frequencies present. The summary from fitting to all values of t is shown in fig. S4(c) and (d). Here, the extracted decay time at each value of t is in the 0–5 ps range, but we are not able to extract the $T_2 = 2$ ps set in the model directly from this. The more interesting information is extracted from the amplitude of each frequency component as a function of

time, t; here we are able to monitor the coherent oscillations of the state populations and their decay over time.

Simplified Two-Level Model

To verify this approach, we repeated the fitting procedure for a simplified 2-level system where the number of energy levels is reduced to 2, so only one transition frequency is present. The parameters for this were kept as given above, except the energy levels, $f_n = 2.3\,\text{THz}$, and the dipole matrix element, $z_{21} = 1\,\text{nm}$. The results of this fitting are shown in figure S5. Examining the amplitude obtained from fitting the nonlinear signal along the τ-axis at each value of t (fig. S5(d)) we recover the coherence lifetime, $T_2 = 2\,\text{ps}$, set in the model.

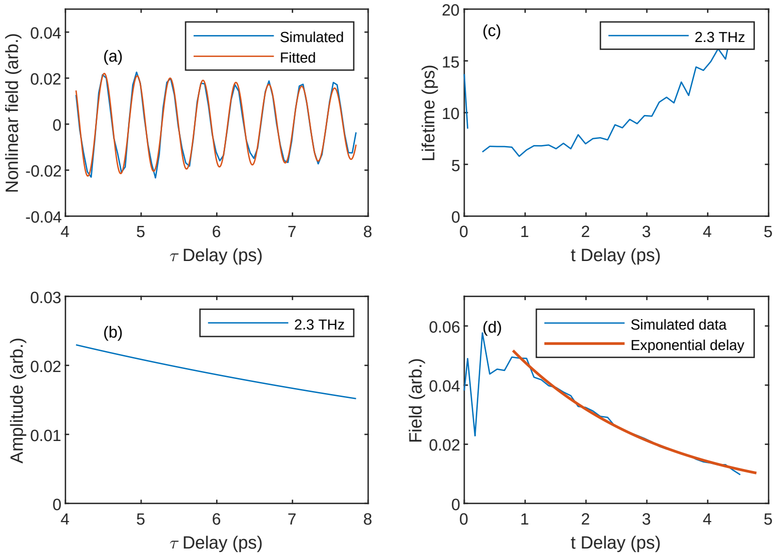

Figure S5: Results of fitting to a 2-level Maxwell-Bloch model. (a) Fitting a slice along the τ-axis for a fixed value of t of the nonlinear signal generated by a 2D-TDS simulation of the 2-level Maxwell-Bloch model. The same equation is used for fitting here as for the 4-level model, but with only one frequency at 2.3 THz. (b) Field envelope from the same fit. (c) Decay lifetime extracted for each value of t, and (d) The maximum value of the field obtained from the fitting as a function of t. The simple exponential fit confirms the coherence lifetime, $T_2 = 2\,\text{ps}$, set in the model.

References

(1) Freeman, J. R.; Maysonnave, J.; Khanna, S.; Linfield, E. H.; Davies, A. G.; Dhillon, S. S.; Tignon, J. Laser-seeding dynamics with few-cycle pulses: Maxwell-Bloch finite-difference time-domain simulations of terahertz quantum cascade lasers. *Phys. Rev. A* **2013**, *87*, 063817.

(2) Ziolkowski, R. W.; Arnold, J. M.; Gogny, D. M. Ultrafast pulse interactions with two-level atoms. *Phys. Rev. A* **1995**, *52*, 3082–3094.

(3) Clauws, P.; Broeckx, J.; E., R.; Vennik, J. Oscillator strengths of shallow impurity spectra in germanium and silicon. *Phys. Rev. B* **1988**, *38*, 12377.
